# A prospective cohort study to assess if alcohol intake measured by routine pregnancy self-report predicts developmental concerns uncovered by routine health visitor screening of children at 30 months of age

**DOI:** 10.1186/s13690-025-01766-2

**Published:** 2025-11-24

**Authors:** David Tappin, Daniel Mackay, Lucy Reynolds, Niamh Fitzgerald

**Affiliations:** 1 Section of Child Health, College of Medical, Veterinary & Life Sciences, Wolfson Medical School Building, University of Glasgow, University Avenue, 31, Shawhill Road, Glasgow, G12 8QQ Scotland, UK; 2 Institute of Health and Wellbeing, Public Health, University of Glasgow, 1 Lilybank Gardens, Glasgow, G12 8RZ Scotland, UK; 3 Institute for Social Marketing & Health, Faculty of Health Sciences & Sport, University of Stirling, Stirling, FK9 4LA UK; 4 SPECTRUM Consortium, London, UK

**Keywords:** Clinical markers, Cohort study, Data collection, Demography, Epidemiology

## Abstract

**Background:**

Stigmatized behaviours are often underreported, especially in pregnancy, making them challenging to address. The Alcohol and Child Development Study (ACDS) seeks to inform prevention of foetal alcohol harm, linking self-report as well as a maternal blood alcohol biomarker with child developmental outcomes.

**Methods:**

Maternity records from all pregnant women in the study city who presented for maternity care during the 12-month period June 2017 – June 2018 were transferred to the safe data facility creating the baseline cohort. Health Visitor routinely recorded child developmental screening data collected when the offspring were 30 months of age were transferred and linkage analysis performed on the cohort with a final linked sample of 10,876 records. Anonymous analysis was performed to assess associations between self-reported alcohol intake collected at maternity presentation with child development concerns and looked after by the local authority status of offspring at 30 months of age. Chi2 tests of association were used with limited multivariate regression to control for confounding variables.

**Results:**

14919 maternity records were transferred and 10,876 could be linked to developmental screening data collected at 30 months of age. As expected self-reported current alcohol use after conception measured at presentation to maternity care was associated with offspring being looked after by the local authority (chi^2^ = 7.85, *p* = 0.005) at age 30 months. 6.6% of children in care were attributable to self-report of current alcohol use during pregnancy. Developmental outcomes were generally inversely associated with self-report of current alcohol use notably concern regarding Social Development (chi^2^ = 4.08, *p* = 0.043) and Speech, Language or Communication Development (chi^2^ = 4.37, *p* = 0.037).

**Conclusions:**

This unexpected inverse relationship could be due to underreporting of alcohol use (the risk) and inaccurate developmental and behavioural problems reported (the outcome) or effective intervention for women who self-report current alcohol intake at maternity presentation.

**Supplementary Information:**

The online version contains supplementary material available at 10.1186/s13690-025-01766-2.

**Table:** 

**Text box 1.** Contributions to the literature
• This paper outlines difficulties addressing heavy alcohol use during pregnancy.
• Rather than alcohol use and measurement thereof being the goal it moves the problem on to adverse outcomes, bypassing arguments of which is the most useful alcohol measure.
• The limitations of maternal self-report to identify at risk alcohol use in order to provide support to stop are highlighted.
• A blood test specific for heavy alcohol use is proposed to allow estimation of the level of misreporting.
• If this blood test predicts poor behavioural and developmental outcomes it may lead to a useful screening test to focus available support services.

## Introduction

Foetal Alcohol Syndrome (FAS) is recognized in the developed world as the leading preventable cause of disorders of intellectual development [1]. According to the literature, the overall combined rate of FAS and Fetal Alcohol Spectrum Disorders (FASD) is estimated to be about 7–18/1,000 births in various populations [2]. Both the diagnosis of FAS/FASD soon after birth [3] and the direct measurement of alcohol in the mother are difficult [4, 5], posing challenges for the evaluation of interventions aimed at reducing dangerous antenatal alcohol consumption. Most cases of developmental impairment that are probably caused by alcohol use during pregnancy are never known to health carers to be related to alcohol use.

A recent review [6] of publications using the Avon Longitudinal Study of Parents and Children (ALSPAC) dataset, a large well recognised longitudinal study which tracked over 14,000 pregnancies from the 1990s, investigated the relationship between prenatal alcohol exposure (PAE) and various offspring outcomes. The review showed mixed results regarding childhood developmental outcomes. Low levels of PAE generally had little measurable effect on childhood developmental outcomes. High levels of PAE and particularly binge drinking lowered IQ. Control for socioeconomic level was important and difficult as women of higher socioeconomic level generally drank more but with less binge drinking. A further cohort study from Australia supports these findings suggesting that low to moderate alcohol use during pregnancy did not effect neurodevelopmental outcomes at early school age and reiterated the importance of socioeconomic status [7]. The Born in Bradford study also indicates that binge drinking (at least five units of alcohol at least once per week after the 4th month of pregnancy) was associated with a lower ‘Good Level of Development’ score at 4–5 years of age [8]. In summary, evidence suggests that high levels of alcohol intake and binge drinking during pregnancy are associated with developmental outcomes that are measureably worse by school age.

Currently all pregnant women in Scotland are asked about alcohol use by their midwife at maternity presentation in early pregnancy [9]. Support offered ranges from a brief intervention [10] to referral for additional support from a specialist nursing team, (who also help women with other known drug addictions), depending on level of alcohol use reported. However, a recent study showed that self-report only identified 38% of women who drank significant alcohol during pregnancy [11]. False negative information is given for a variety of reasons: social stigma, inaccurate recollection and problems estimating alcohol content or volume consumed with consequent under-reporting [12]. Self-report is also influenced by a desire to provide socially acceptable information [13] or for fear of censure and intervention by social services [14]. Other treatable conditions that might be hidden or unknown like smoking and hepatitis B can be tested for using a breath test and blood test respectively, so that all can be offered effective therapy or treatment. There is currently no recognised test to augment self-report of alcohol use during pregnancy. This means that specific treatment for the mother, her child and prevention of damage for future children is often not provided.

The Alcohol and Child Development Study (ACDS) includes a full annual cohort of pregnant women in a UK city and aims to inform the development of improved methods of identification of women at risk of having a child affected by foetal alcohol exposure. The study aims to follow anonymously the children from this cohort of women identified at their first maternity visit. Examination for a relationship will be made between self-reported alcohol consumption in pregnancy and/or a positive maternal blood test for an alcohol biomarker and later childhood developmental impairment. Developmental impairments have been taken from routine health visitor developmental screening at 30 months of age and will also be derived from routine clinical assessment of any of the cohort of children referred for suspected developmental problems requiring specialist assessment and treatment pre-school.

This paper reports self-report of alcohol use before and during early pregnancy, and referral for support. Associations with developmental concerns discovered during routine developmental screening by health visitors at 30 months postnatal age are reported.

## Methods

### Design

This paper compares routinely collected data related to alcohol use before and during pregnancy from women attending ‘booking’ appointments for maternity care in the study city, with later routine screening child development information collected at 30 months of age by health visitors.

### Setting

The setting for the ACDS was midwife-led first maternity clinic appointments at 13/14 weeks of pregnancy in a medium sized UK city between June 2017 and June 2018 (‘the study period’). These appointments are to start the midwife-led pregnancy support programme and include a formal pregnancy test, breath test for smoking as well as routine blood tests for infection with hepatitis B, syphilis and HIV for which current treatment improves birth outcomes. In Scotland, women are also asked about alcohol use. If significant alcohol consumption is reported, women are provided with a brief intervention or referral to specialist services to support alcohol reduction and elimination during pregnancy.

### Participants and information provided

All pregnant women reporting to the NHS in the study city during the study period received a study information sheet (PIS) along with the letter informing them of their booking appointment date and time and all women who attended during the analysis period are included in the analysis for this paper as outlined above. In line with the ethical approval conditions, the PIS informed women that the NHS wished “to collect an extra 2 ml (one third of a teaspoon) of blood from all pregnant women”, and that this extra blood would be “used anonymously for research … which may help us to learn more about the best ways to support the health of women and babies and improve our service.” Women were informed that they could opt out of the study by letting their midwife know that they did not want the sample taken. A contact name and number was provided on the PIS for any queries.

Similar generic information was provided to all midwives by a senior research midwife unconnected to the study team, and they were advised on when and how to take the additional sample. Midwives were asked not to actively consent the women before taking the extra sample, but to take the sample ‘routinely’ unless the woman (from reading the PIS sent with the booking appointment notification) voiced a wish to opt out. The potential testing of the additional blood sample for an alcohol biomarker was not disclosed to women or frontline midwives.

The blood samples were stored in a sample repository with a study identifier linked to routine maternity booking data held by a secure NHS data service such that neither the researchers nor clinicians could identify who had a sample taken. The study aimed to assay these samples for a marker currently used by the Driver and Vehicle Licensing Agency (DVLA) (https://www.drinkdriving.org/cdt-alcohol-test.php) to assess current heavy alcohol intake– Carbohydrate Deficient Transferrin - CDT [15]. This marker allows assessment of heavy alcohol use during the preceeding 7 days and gives the DVLA a marker of current alcohol use, when a recurrent alcohol related offender applies to have their driving licence reinstated. This article does not discuss the results from the CDT tests as the samples are currently being assayed.

### Quantitative variables and data sources

Detailed self-report of alcohol use was collected at the first maternity visit via a locally developed computerised data system until 31 st October 2017. From November 2017 the computer system was changed to a Scotland wide system that potentially meant less detailed self-reported alcohol use information during pregnancy was recorded, through to the end of sample collection in June 2018. With the exception of birth weight of the baby, variables were gathered from routinely collected maternity service data recorded directly by midwives onto the computerised database. Only those variables which were deemed not to risk the anonymity of participants were shared with researchers. Shared data included the following:

- Area-based material deprivation [16], calculated from patient postcode using government statistics.
- Age
- Height and weight at the time of booking, used to calculate Body Mass Index (BMI) using the formula Weight (in kilos)/Height2 (in metres).
- Number of previous pregnancies, previous spontaneous abortions, previous therapeutic abortions.
- Smoking history, self-reported smoking while pregnant, carbon monoxide breath test level.
- Estimated gestation in weeks calculated from recall of last menstrual period, and.
- Self-reported alcohol use. This was recorded in 16 domains, of which just four of these contained sufficient data for the safe data facility to be sure that anonymity would be retained. How much did you drink daily before pregnancy? (converted into alcohol units); Brief Intervention was required Yes/No?; Referral to (alcohol) intervention nurse Yes/No?; How much do you drink each week now or since conception? (converted into alcohol units).
- The birth weight of the baby was obtained through record linkage with a national database.

All of this data was transferred to and held by an NHS safe data facility to enable future linkage with child health records without compromising the anonymity of the participants or their children.

### Outcome data

The universal pathway for preschool children in Scotland includes the offer of an in-person health visitor contact by Health Visitors gathering data from parents/carers at 27 to 30 months postnatal age [17, 18]. This usually takes place in the child’s home, with a parent/carer as informant. As part of that contact, the Health Visitor reviews information already available in the child’s record, listens to any new concerns the family may have, and undertakes developmental observations. Data items recorded include: Looked After Child status (information already known by the Health Visitor); Carer’s Smoking status; Child Exposed to ETS (Environmental Tobacco Smoke); and developmental status in fields of Social Development; Emotional Development; Speech, Language and Communication Development; Gross Motor Development; Fine Motor Development; Vision; Hearing. Responses are recorded as: New concern; Previous concern; No concern; No meaningful result. Whilst in the sensory and motor fields threshold for recording concern was a matter of professional judgement, assessment of social and emotional development and communication skills was supported by two standardized tools universally employed in the Health Board area at the time of the study: the Surestart Language Measure – Revised (SSLM-R) and the Strengths and Difficulties Questionnaire (SDQ). Threshold for possible concern on the SSLM-R was if the child was using fewer than 32 of the 50 words listed (or there were concerns about comprehension, stammering or stumbling over words). For the SDQ it was a Total Difficulties score of 17 or above. The Ages and Stages Questionnaire (ASQ) has now been rolled out across Scotland, but was not in general use at the time of the study. Health Visitors complete a 27–30m assessment template on a computer-based data collection system (EMIS) and then this data is extracted to the national data set [18]. Data held by the health board for children born between 1st August 2017 and 28th February 2019 were provided to the safe data facility to include pregnancies where women booked for maternity care between 12th June 2017 and 30th June 2018. Most data was collected at 30 months postnatal age during 2020 when the percentage of children from whom collection was complete are shown below (Table 1).

**Table 1:** Percentage of children where 27–30 review data were collected by calendar month for 2020

Month	Jan	Feb	Mar	Apr	May	June	Jul	Aug	Sep	Oct	Nov	Dec
**%**	94%	93%	91%	85%	72%	57%	65%	76%	84%	88%	90%	92%

*Note.* Information was made available by the city wide health visiting service.

### Bias

The unusual consent procedures in this study described above – passive consent for generic research using a routine extra blood sample - were designed to minimise sources of bias in the cohort. Bias and ethical issues have been discussed previously [19].

### Study size

The whole cohort from 12th June 2017 to 30th June 2018 was chosen to include all festivals and holiday periods during a full calendar year when alcohol intake may increase.

### Statistical methods

Statistical analyses were performed with Stata 12.10 [20]. Frequencies of levels of alcohol use on a daily basis pre-pregnancy and on a weekly basis since known start of pregnancy (end of last menstrual period) recorded in computer based antenatal questionnaire records are described. Actions by maternity staff to questionnaire responses including brief alcohol interventions and referral to Specialist Nurse-led services for alcohol and drug addiction are described. A new ‘Current Alcohol Use’ variable was developed using any alcohol on a weekly basis versus no alcohol since the start of pregnancy (Yes/No). Where information on alcohol use since the start of pregnancy was missing, any alcohol use on a daily basis prior to pregnancy if recorded (Yes/No) was used instead. The rationale was: in the absence of specific self-report of weekly alcohol use after the start of pregnancy, we decided that daily alcohol habit before pregnancy would probably have continued into the first days and perhaps weeks prior to pregnancy being known.

Local maternity unit guidance is that if a pregnant woman indicates that she is currently drinking 15 units of alcohol per week or more, a referral to the nurse led service for specialist help during pregnancy is required; for 1–14 units an Alcohol Brief Intervention should be offered by the attending midwife.

Frequency of outcomes from 30-month developmental assessment by health visitors (supplementary Fig. 1) are described.

Maternity records held in the safe data facility were linked to the child’s records through the child vaccination record which includes the maternal Community Health Index (CHI) number. To those pregnancy records held in the safe data facility, where a birth and vaccination record for a baby had been generated, a child CHI number was added. 30-month routine health visitor developmental assessment was linked to maternity records held in the safe data facility using the child’s CHI number.

Univariate analysis of association using crosstabulation of the new alcohol variable ‘Current Alcohol Use’ and individual outcomes as well as a combined variable (any developmental concern) from the 30-month health visitor assessment used Pearson X2 for significance testing. Multivariate logistic regression was used to control for potential confounding variables recorded when statistical power was adequate. Population attributable risk percent for children being Looked After by the local authority was calculated from the Attributable Risk and the proportion of children Looked After by the local authority in the whole population of children in whom data was collected.

### Ethics

Ethics approval was granted by the local NHS Research Ethics Committee (28/10/2010 - approval number provided, but not published to protect identity of the study site). This was previously discussed in detail [19]. Passive consent was utilised in an attempt to remove bias by making the collection of extra blood at the time of routine venapuncture as straightforward as possible for both maternity staff and patients in busy first maternity visit clinics.

The sponsor for study was the local NHS management authority.

## Results

14,919 maternity records were transferred, 13,430 could be linked to a live child through vaccination records and 10,876 could be linked to developmental screening data collected at 30 months of age. Self-reported alcohol use data for the cohort of pregnant women who booked for maternity care (and delivered a live newborn (*n* = 13430) so an infant CHI number was generated) in the city between 17th June 2017 and the 30th June 2018 is shown in Table 2. How much did you drink daily before pregnancy? (converted into alcohol units); Brief Intervention was required Yes/No?; Referral to (alcohol) intervention nurse Yes/No?; How much do you drink each week now? (converted into alcohol units). Only these variables had sufficient data to be analysed as discussed previously [19]. It is interesting to note that local guidance where current alcohol use greater than zero and less than 15 units per week should prompt a brief intervention my midwifery staff has largely not taken place as only 31 brief interventions are documented for 526 women in this group. This may reflect lack of time, private place to provide the intervention or confidence of midwifery staff to follow through with this task.

**Table 2:** Alcohol consumed before and during pregnancy and intervention given for reported alcohol use collected at first maternity visit

Alcohol units per day before conception		
Units	Frequency	Percentage
0	10,724	79.9
1–4	1247	9.3
5–14	90	0.7
>=15	21	0.2
Not asked or not known	1348	10.0
Total	13,430	100.0
Alcohol units per week now/since conception		
0	12,099	90.1
1–4	402	3.0
5–14	124	0.9
>=15	22	0.2
Not asked or not known	783	5.8
Total	13,430	100.0
Brief intervention given		
Yes	31	0.2
No	13,399	99.8
Total	13,430	100.0
Referred to alcohol intervention nurse/team		
Yes	246	1.8
No	13,184	98.2
Total	13,430	100.0

Frequencies of observations for 27–30 month developmental assessment are shown in supplementary Table 1 for 10,876 children where data was recorded. There were good levels of completion (92–99%) for nearly all variables examined which were: Looked After Child status; Carer’s Smoking status; Child Exposed to Environmental Tobacco Smoke; Social Development; Emotional Development; Speech, Language and Communication Development; Gross Motor Development; Fine Motor Development; Vision; Hearing. Ages and Stages Questionnaire (ASQ) was only completed for 428(4%) of children as a pilot for future routine use and was not examined further.

Cross-tabulation of these outcomes with ‘Current Alcohol Use’ showed that Social Development (chi2 = 4.08, *p* = 0.04) Table 3 had a greater proportion of concerns among women where *no* alcohol use was reported during pregnancy, as did concerns regarding Speech, Language, Communication Development (chi2 = 4.37, *p* = 0.04) Table 3.

**Table 3:** Crosstabulation of ‘Current alcohol use’ collected routinely at first maternity visit against developmental concerns recorded by health visitors at 30 month routine developmental screening

Current alcohol use	Developmental concern collected at 30 months of age			
	Yes	No	Total	Pearson chi2
	*Social Development*			
Yes	17(3.9%)	425(96.1%)	442(100%)	4.08 *p* = 0.04
No	589(6.2%)	8914(93.8%)	9503(100%)	
Total	606	9338	9945	
	*Speech*,* Language or Communication Development*			
Yes	54(12.2%)	387(87.8%)	441(100%)	4.38 *p* = 0.04
No	1507 (15.9%)	7931(84.1%)	9437(100%)	
Total	1561	8318	9879	
	*Any Developmental concern*			
Yes	83(14.6%)	329(85.4%)	412(100%)	1.85 *p* = 0.18
No	2050(17.0%)	6854(83.0%)	8904(100%)	
Total	2133	7183	9316	
	*Emotional Development*			
Yes	28(6.3%)	414 (93.7%)	442(100%)	2.11 *p* = 0.15
No	788(8.3%)	8728(91.7%)	9516(100%)	
Total	816	9142	9958	
	*Gross Motor Development*			
Yes	< 5(0.0–1.0%)	438(99%−100%)	(100%)	3.08 *p* = 0.08
No	171(1.8%)	9317(98.2%)	9488(100%)	
Total		9755		
	*Fine Motor Development*			
Yes	< 5(0.0–1.0%)	438(99%−100%)	(100%)	1.69 *p* = 0.19
No	105(1.1%)	9358(98.9%)	9463(100%)	
Total		9796		
	*Hearing Development*			
Yes	5(1.2%)	403(98.8%)	408(100%)	1.58 *p* = 0.21
No	190(2.1%)	8705(97.8%)	8896(100%)	
Total	195	9109	9304	
	*Vision Development*			
Yes	12(2.9%)	399(97.1%)	411(100%)	0.59 *p* = 0.44
No	208(2.3%)	8708(97.7%)	8916(100%)	
Total	220	9107	9327	

*Note.* %ages are row %ages and rows refer to those with ‘current alcohol use’ versus those without

‘Any Developmental Concern’ showed no-significant association (chi2 = 2.19, *p* = 0.14) Table 3 and when subjected to multiple logistic regression the odds ratio of there being a developmental concern with ‘Current Alcohol Use’ was 0.80 (95%CI 0.62, 1.03 *p* = 0.08), in the direction of *fewer* concerns. Emotional Development (chi2 = 2.11, *p* = 0.147), Gross Motor Development (chi2 = 3.08, *p* = 0.08), Fine Motor Development (chi2 = 1.69, *p* = 0.19), Hearing Development (chi2 = 1.58, *p* = 0.21) all were in the direction of less concerns with a positive self-report of ‘Current Alcohol Use’. Vision Development (chi2 = 0.59, *p* = 0.44) was not statistically significant but was in the direction of more concerns for those self-reporting ‘Current Alcohol Use’.

The only data item with an ‘expected’ [21] statistically significant association with ‘Current Alcohol Use’ was Looked After (by the local authority) shown in Table 4.

**Table 4:** Crosstabulation of ‘Current alcohol use’ collected at first maternity visit against looked after by the local authority status documented by health visitors at 30 month routine developmental screening contact

Current Alcohol Use	Looked After (by the local authority) at 30 months of age			
	Yes	No	Total	Pearson Chi2
Yes	9 (2.0%)	439(98.0%)	448(100%)	7.85 *p* = 0.005
No	75 (0.8%)	9563(99.2%)	9638(100%)	
Total	84	10,002	10,086	

*Note.* %ages are row %ages and rows refer to those with ‘current alcohol use’ versus those without.

Incidence of being ‘Looked After’ in whole population = 84/10,085.

Attributable Risk = a/(a + b) – c/(c + d) = 9/448–75/9637 = 0.0123.

PAR = AR x P_e_ = 0.0123 × 448/10,085.

PAR% = PAR/Incidence of Looked after x 100 = (0.0123 × 448/10085)/(84/10085) = 6.6%.

The population attibutable risk calculation suggests that for about 7% of children looked after by the local authority this is attributable to self-report of current alcohol use at presentation for maternity care.

Smoking during pregnancy and children exposed to environmental tobacco smoke had increased risk of ‘any developmental concern’ by 56% (*p* < 0.001) and 59% (*p* < 0.001) respectively. Low birthweight (< 2500 g) babies were 2.7 times (*p* < 0.001) more likely to have a concern than those of normal weight. The most affluent areas (Scottish Index of Multiple Deprivation SIMD10) [16] had a 63% lower risk compared with the most deprived (SIMD 1) (*p* < 0.001) and boys were twice as likely as girls to be recorded as having ‘any developmental concern’ (*p* < 0.001).

## Discussion

### Summarised study results

This study suggests that self-report of ‘Current Alcohol Use’ disclosed at maternity booking, usually in the early second trimester of pregnancy, was inversely associated with concerns about some developmental outcomes collected by health visitors from parents at 30 months postnatal age. The study suggests that local guidance to offer intervention to women who report current alcohol use at the first maternity visit was not consistently adhered to.

### Previous research

Findings regarding child development are surprising as they contradict most other evidence that maternal alcohol use during pregnancy is detrimental to child development outcomes [22, 23]. In contrast, other variables including tobacco smoking, low birth weight, male gender and material deprivation were all associated with an increase in ‘any developmental concern’.

### Discussion of results

There are a number of possible reasons for these interesting findings that can be split into 3 categories:

1. Measurement of ‘risk’: Maternal alcohol use collected at first maternity visit by ‘self-report’ is known to misreport many women who drink alcohol during pregnancy [11] because of perceived fear of judgement by health service staff and even social services intervention to protect the child once born. This is backed up by the only outcome not relying on maternal self-report, ‘Looked-after’ status, which shows an ‘expected’ positive relationship with self-reported alcohol use during pregnancy.
2. Measurement of ‘outcome’: Health Visitor led developmental assessment at 30 months postnatal age, although structured, relies in part on parental self-report which may be inaccurate at predicting poor outcomes. ‘Effectiveness of screening’ assessment for this type of child development screening pre-school is yet to be undertaken. Families of up to 10% of children do not accept the offer of this developmental screening contact [24] and the most in need are likely to be over-represented in the ‘unscreened’. These results were also collected during COVID when some screening was undertaken by telephone relying only on parental self-report rather than the usual norm of direct observation by Health Visitors.
3. There is an inverse association between the frequency of some developmental concerns at 30 months and self-reported ‘Current Alcohol Use’ documented at maternity booking perhaps because pregnant women who self-report alcohol use receive an effective intervention (either a brief intervention from midwifery staff for any reported current alcohol intake or more extensive support from specialist nurses if current reported alcohol intake is high). Brief interventions were seldom documented to have taken place (only 31 brief interventions for 526 women who reported current alcohol intake of greater than 0 units per week and less than 15 units per week – Table 2). However many more referrals to Specialist Nursing Services for alcohol and drug use took place than women who reported ‘Current Alcohol Use’ greater than 15 units per week (22 reports versus 246 referrals). These additional referrals may have been for other issues such as drug addiction or because midwifery staff felt that providing another source of support was appropriate. Whoever provided support, drinking alcohol may have stopped for the rest of these pregnancies and developmental concerns in toddlers could have therefore been averted. This possibility is supported by those not reporting current alcohol use having significantly more developmental ‘concerns’ particularly related to ‘social development’ and ‘speech language or communication development’ which are areas often reported with alcohol related damage [25]. Those who self-report as no current alcohol use will include a proportion of women who are ‘misreporting’ and are in fact current alcohol users at maternity booking. These women will therefore not receive an intervention to stop drinking alcohol at maternity presentation, either a brief intervention or referral to the specialist nursing team for drug and alcohol use, whereas those who self-report current alcohol use are offered an intervention and may therefore stop drinking alcohol.

In order to resolve possibility 1., a better method of documenting excessive alcohol use preferably before pregnancy, but at least early in pregnancy is required. This study has collected extra blood samples to test anonymously for an alcohol marker at maternity booking. Once these samples are assayed, the results can be linked in the safe data facility to self-report of current alcohol use and developmental outcome measures.

To resolve possibility 2., a better outcome measure would be referral (often from health visitors or pre-school nursery placement) for assessment by specialist community-based child health services including: speech and language therapists, specialist nurses and paediatricians in child development teams, physiotherapists, occupational therapists, hearing and vision assessment, psychologists, psychiatrists or the health team involved in fostering and adoption services. Observation over time in a nursery setting which graduates children towards successful school placement misses few children who require further assessment and support. Referrals are made in as timely a way as possible to facilitate children receiving help and assessment prior to primary school placement. Documentation of referrals and subsequent assessments by community services are held on one local EMIS database. Linkage of the study cohort with outcomes up to school age is being undertaken to assess the sensitivity and specificity of the 27–30 month developmental screening undertaken by Health Visitors.

For the third possibility, if the new developmental outcome discussed above, ‘referral for assessment and treatment of developmental and behavioural concerns’ was in general agreement with 30-month developmental screening by health visitors, then it would suggest that obstetric staff are utilising an *effective intervention strategy* to reduce self-reported alcohol use at maternity booking. It would therefore become more pressing to identify all pregnancy alcohol use at maternity presentation and to fully implement interventions when women do self-report current alcohol use. By testing the stored serum samples for Carbohydrate Deficient Transferrin (which has low sensitivity for any alcohol use but high specificity for heavy alcohol use), the size of under-reporting of current alcohol use at maternity booking in this cohort of pregnant women can be estimated.

### Study limitations

The main study limitation is the lack of power to employ multivariate modelling in order to control for potentially confounding variables. The most important of these is socioeconomic status as it is known that women of high socioeconomic status tend to drink more alcohol during pregnancy but usually do not ‘binge’ drink [6]. High socioecomic status is associated with less developmental concerns which may mask detrimental effects of alcohol use during pregnancy.

### Study implications

This study implies that current methods to discover current alcohol use at maternity booking and to provide active intervention (brief intervention and/or specialist nursing support) in many cases miss the chance of providing appropriate support. This is due to misreporting of current alcohol use and to difficulties implementing the guidance for a planned intervention when current alcohol use is reported. In the past the local maternity services had a similar problem with smoking during pregnancy. NICE guideline PH26 2010 [26], supported screening of all pregnant women for carbon monoxide level using a breath test which largely eliminated the problem of misreporting current smoking habit. PH26 also suggested that specialist services for smoking cessation should be utilised where busy maternity services were not able to provide consistent support. There is a local specialist nursing service for women who report current heavy alcohol use at first maternity visit but a screening system to resolve the problem of misreporting has yet to be developed.

### Directions for future research

This new developmental outcome, ‘referral for assessment and treatment of developmental and behavioural concerns’ is currently being examined. Data for the study cohort has been transferred to the safe data facility, linked to the study cohort and is awaiting anonymous analysis. Funding to assay the remaining serum samples for Carbohydrate Deficient Transferrin (CDT) has been secured. If CDT results from maternity booking were to predict development and behavioural difficulties often assessed prior to school placement and intervention to reduce alcohol consumption initiated at maternity booking was seen to reduce these difficulties, then screening for CDT at maternity booking, similar to carbon monoxide breath testing for smoking and blood screening for hepatitis B and syphilis, could be considered. Such a screening programme throughout the UK would not be undertaken unless it could be proved that detection of misreporting of heavy alcohol use by CDT and full implementation of intervention strategies reduced subsequent developmental and behavioural problems in offspring and improved the health of the pregnant population.

## Conclusion

This paper underlines the difficulty uncovering current alcohol use at first maternity visit in order to offer support to stop. It also highlights that even when current alcohol use is reported at first maternity visit, support is not always provided. Specialist nursing services are available locally and could be expanded to provide support for all women who report current alcohol use. A screening test for heavy alcohol use at the first maternity visit has yet to be implemented. If raised Carbohydrate Deficient Transferrin was shown to predict developmental problems known to be associated with heavy alcohol use during pregnancy, then it’s use as a screening test should be considered.

## Supplementary Information

Supplementary Material 1.

## Data Availability

Data from this study is not available for others to re-analyse as the data is held behind a Safe Data facility. Analysis can only be undertaken on the Safe Data facility platform where results can be checked before release so that inadvertent identification of individuals does not take place.
