# Supplementary material for: A prospective cohort study to assess if alcohol intake measured by routine pregnancy self-report predicts developmental concerns uncovered by routine health visitor screening of children at 30 months of age
Source: Arch Public Health. 2025 Nov 24;83:283. doi: 10.1186/s13690-025-01766-2 (PMC12642173; doi:10.1186/s13690-025-01766-2)

**Supplementary Table 1**

*Frequency of outcomes collected at the 30 month developmental assessment by health visitors*

| **Looked after by the local authority** | **Freq** | **Percentage** |
| --- | --- | --- |
| Not Looked After | 10764 | 98.9 |
| Looked after | 95 | 0.9 |
| Not known | 17 | 0.2 |
| **Carer’s smoking status** |  |  |
| Non-smoker | 9515 | 87.5 |
| Smoker | 1352 | 12.4 |
| Not known | 9 | 0.1 |
| **Child exposed to exhaled tobacco smoke (ETS)** | | |
| No ETS exposure | 10157 | 93.4 |
| ETS exposure | 709 | 6.5 |
| Not known | 10 | 0.1 |
| **Social Development** | | |
| New concern | 415 | 3.8 |
| Previous concern | 239 | 2.2 |
| No concern | 10059 | 92.5 |
| No meaningful result | 163 | 1.5 |
| **Emotional Development** | | |
| New concern | 625 | 5.8 |
| Previous concern | 251 | 2.3 |
| No concern | 9848 | 90.6 |
| No meaningful result | 152 | 1.4 |
| **Speech, Language and Communication Development** | | |
| New concern | 1225 | 11.2 |
| Previous concern | 447 | 4.1 |
| No concern | 8964 | 82.4 |
| No meaningful result | 240 | 2.2 |
| **Gross Motor Development** | | |
| New concern | 65 | 0.6 |
| Previous concern | 120 | 1.7 |
| No concern | 10508 | 98.3 |
| No meaningful result | 183 | 1.7 |
| **Fine Motor Development** | | |
| New concern | 54 | 0.5 |
| Previous concern | 68 | 0.6 |
| No concern | 10542 | 96.9 |
| No meaningful result | 212 | 2.0 |
| **Vision Development** | | |
| New concern | 76 | 0.7 |
| Previous concern | 163 | 1.5 |
| No concern | 9814 | 90.2 |
| No meaningful result | 823 | 7.6 |
| **Hearing Development** | | |
| New concern | 99 | 0.9 |
| Previous concern | 105 | 1.0 |
| No concern | 9827 | 90.4 |
| No meaningful result | 845 | 7.8 |
| Total | 10876 | 100.0 |

**Supplementary figure 1: 30 months developmental assessment data collection tool**


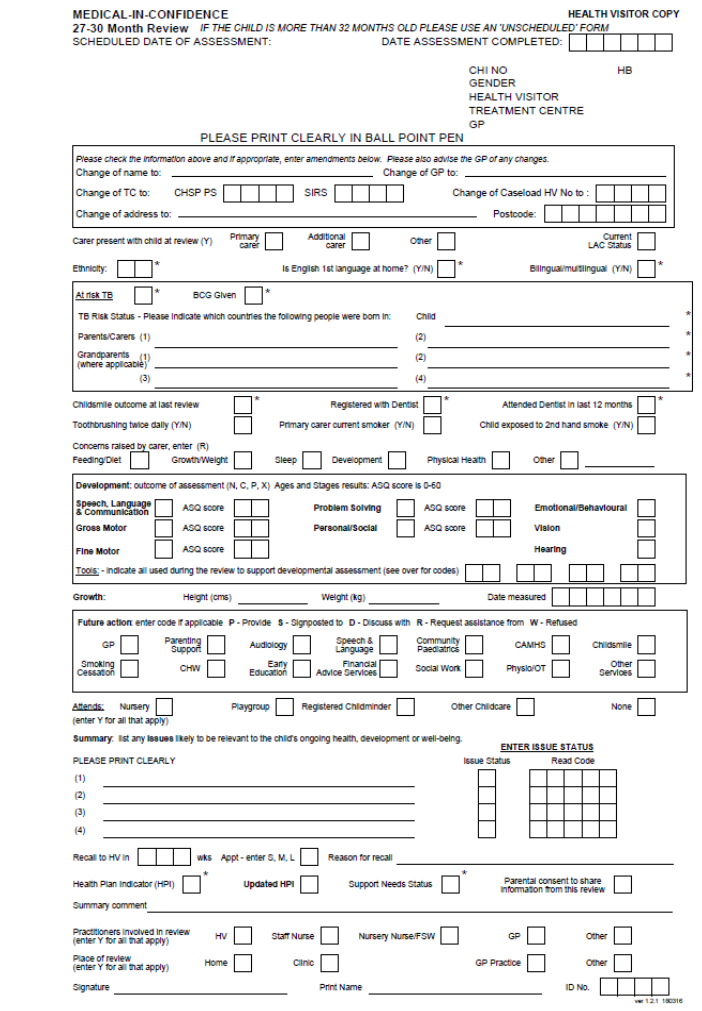

Supplement: Supplementary file 1 — Supplementary Material 1. [file 13690_2025_1766_MOESM1_ESM.docx]
